# Interrupting the Conversation: Implications for Crosstalk Between Viral and Bacterial Infections in the Asthmatic Airway

**DOI:** 10.3389/falgy.2021.738987

**Published:** 2021-10-26

**Authors:** Jodie Ackland, Alastair Watson, Tom M. A. Wilkinson, Karl J. Staples

**Affiliations:** ^1^Clinical and Experimental Sciences, University of Southampton Faculty of Medicine, Southampton, United Kingdom; ^2^NIHR Southampton Biomedical Research Centre, University Hospital Southampton NHS Foundation Trust, Southampton, United Kingdom; ^3^College of Medical and Dental Sciences, University of Birmingham, Birmingham, United Kingdom; ^4^Wessex Investigational Sciences Hub, University of Southampton Faculty of Medicine, Southampton General Hospital, Southampton, United Kingdom

**Keywords:** asthma, bacteria, virus, co-infection, exacerbation, inflammation, early-life

## Abstract

Asthma is a heterogeneous, chronic respiratory disease affecting 300 million people and is thought to be driven by different inflammatory endotypes influenced by a myriad of genetic and environmental factors. The complexity of asthma has rendered it challenging to develop preventative and disease modifying therapies and it remains an unmet clinical need. Whilst many factors have been implicated in asthma pathogenesis and exacerbations, evidence indicates a prominent role for respiratory viruses. However, advances in culture-independent detection methods and extensive microbial profiling of the lung, have also demonstrated a role for respiratory bacteria in asthma. In particular, airway colonization by the Proteobacteria species Nontypeable *Haemophilus influenzae* (NTHi) and *Moraxella catarrhalis* (Mcat) is associated with increased risk of developing recurrent wheeze and asthma in early life, poor clinical outcomes in established adult asthma and the development of more severe inflammatory phenotypes. Furthermore, emerging evidence indicates that bacterial-viral interactions may influence exacerbation risk and disease severity, highlighting the need to consider the impact chronic airway colonization by respiratory bacteria has on influencing host responses to viral infection. In this review, we first outline the currently understood role of viral and bacterial infections in precipitating asthma exacerbations and discuss the underappreciated potential impact of bacteria-virus crosstalk in modulating host responses. We discuss the mechanisms by which early life infection may predispose to asthma development. Finally, we consider how infection and persistent airway colonization may drive different asthma phenotypes, with a view to identifying pathophysiological mechanisms that may prove tractable to new treatment modalities.

## Introduction

Asthma is a complex and heterogeneous disease of the airways characterized by episodic and reversible airway obstruction, bronchial hyper-responsiveness, and airway inflammation that affects over 300 million people globally (1). Asthma is diagnosed by assessment of airway reversibility performed by spirometry before and after bronchodilator use (2). Allergic sensitivities are a risk factor for asthma development and can be tested for using the skin prick test, which measures reactions to a variety of common environmental allergens (3), or measuring serum levels of IgE (4). However, non-allergic (or non-atopic) forms of asthma can develop following exposure to a non-allergic environmental trigger. As such, clinically defining and treating asthma is complex due to a number of clinical asthma phenotypes displaying different disease pathologies, which are further underpinned by multiple inflammatory endotypes (5, 6).

**Figure 1:**
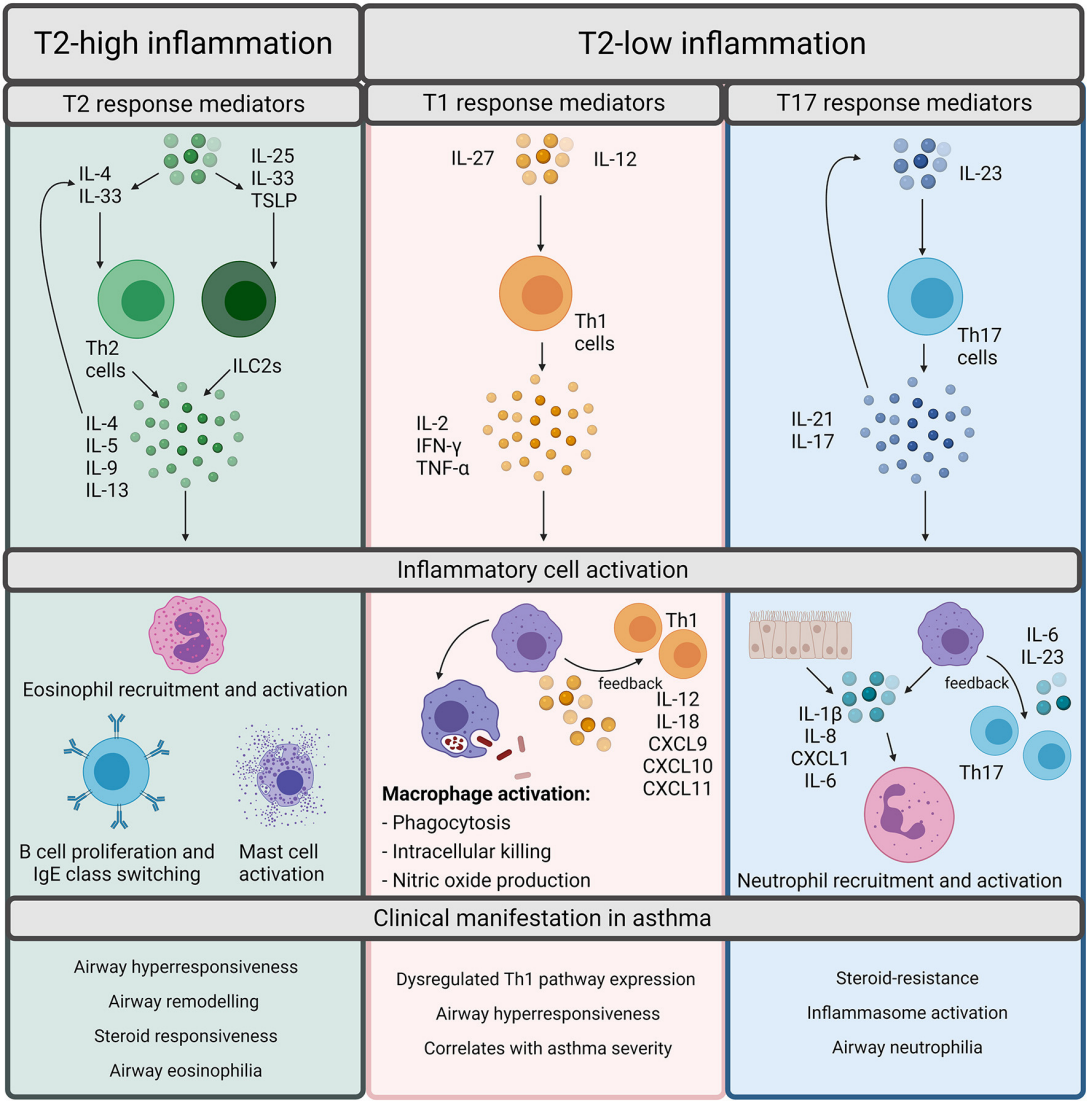
Overview of inflammatory pathways in asthma. Inflammation in asthma is defined as Type (T)2-high or T2-low. T2-high inflammation is characterized by T2 cytokines such as interleukin (IL)-4, IL-5, IL-9, and IL-13. These cytokines can be released by T helper (Th) 2 cells or innate lymphoid group 2 cells (ILC2s). Th2 cells are stimulated by IL-4 whereas ILC2s are stimulated by IL-25, IL-33, or Thymic Stromal Lymphopoietin (TSLP). The T2 cytokines promote the cellular features of T2 inflammation including eosinophil recruitment and activation, B cell proliferation, IgE class switching, and mast cell activation. Although T2 inflammation is generally responsive to steroids, it results in the classical pathophysiological features of asthma including airway hyperresponsiveness, remodeling, and eosinophilia. T2-low responses are often associated with T1 or T17 inflammation. T1 inflammatory responses are regarded as important for defense against intracellular pathogens and are driven by Th1 cells, which are activated upon stimulation by either IL-27 or IL-12. Th1 cells release IL-2, IFN-γ, or TNF-α which activates macrophages and promotes microbial killing. Macrophages also participate in positive feedback through production of IL-12/IL-18, CXCL9, CXCL10, and CXCL11 to amplify T1 inflammation. T17 responses are induced through IL-23 stimulation of Th17 cells. Th17 cells release IL-21 and IL-17 which can either autoregulate Th17 cell differentiation or can act upon epithelial cells or macrophages to release IL-1β, IL-8, CXCL1, and IL-6 to promote neutrophil recruitment and activation. T17 responses are associated with the neutrophilic asthma phenotypes, inflammasome activation, and steroid-resistance in asthma. Created using BioRender.com.

Asthma phenotypes are broadly split into allergic and non-allergic (5), with allergic asthma being the most widely recognized form and implicated in 50–80% of asthma cases (7). Allergic asthma is induced by common environmental allergens including house dust mite, grass and tree pollen, mold and ragweed and is characterized by type 2 (T2) inflammation (5). T2 inflammation is driven by cytokines such as interleukin (IL)-4, IL-5, and IL-13 released by T helper (Th) 2 cells which increase the recruitment and survival of eosinophils (Figure 1). T2 inflammation promotes the pathophysiological features of eosinophilic asthma including increased basement membrane thickness and corticosteroid responsiveness (5, 6, 8–10). However, an increasing number of studies demonstrate evidence of T2-low inflammation in some asthma patients (11, 12). T2-low responses are associated with T1 or T17 inflammation, increased inflammasome responses and corticosteroid insensitivity [Figure 1; (13)]. In contrast to T2-high and eosinophilic inflammation, a major feature of T2-low inflammation is increased neutrophil infiltration. Neutrophils are now understood to play a significant role in asthma and are associated with severe asthma phenotypes, severe airflow obstruction, steroid-resistance and increased presence of potentially pathogenic bacteria (14–16). However, there are individuals who exhibit high levels of both eosinophils and neutrophils, designated as a mixed granulocytic phenotype, which is also associated with more severe asthma and poor responses to current treatments, despite the presence of eosinophils (17).

The multifactorial nature of asthma is such that therapies concentrate on decreasing symptoms rather than disease cure. The most commonly used therapies include inhaled/oral corticosteroids and leukotriene modifiers to control airway inflammation and bronchodilators such as β_2_-agonists or anticholinergics for immediate relief of asthma symptoms (18). However, even patients with good treatment adherence can still experience exacerbations of their asthma symptoms (19). An asthma exacerbation is characterized by a worsening of symptoms and can be graded in severity as mild, moderate, severe or life-threatening (20). Symptoms include wheeze, shortness of breath and chest tightness and are induced by airway inflammation, resulting in airflow obstruction and increased airway responsiveness (2, 21). With ~65,000 hospital admissions yearly in the UK, exacerbations contribute to the considerable financial and logistical burden of asthma on the health care system, with the added economic impact due to lost productivity of workers (22). Exacerbations can also enhance disease progression by increasing the rate of lung function decline and thus asthma severity (23–25), with some more severe forms of asthma resistant to even high doses of steroid treatment (10).

The future of asthma treatment and management is moving towards modulating specific components of the immune system involved in asthma pathogenesis by use of monoclonal antibodies targeting specific inflammatory mediators (26, 27). As such, a number of therapeutics aim to reduce the number of eosinophils and T2 cytokines using anti-IL5 antibodies. However, the mechanisms driving efficacy of anti-IL5 antibodies is unclear: although blood eosinophils are reduced, the impact on airway responses, such as sputum eosinophils, exacerbation frequency, and pulmonary function is varied (28). Importantly, even individuals treated with the anti-IL5R monoclonal antibody, benralizumab, have been reported to experience exacerbations that were predominantly non-eosinophilic in nature and associated with infection (29). Thus, given the mixed outcomes of targeting IL-5/eosinophils and the potential for the involvement of other inflammatory immune cells in asthma pathogenesis, there remains an unmet need for better asthma treatments, particularly in terms of exacerbation prevention (30). Triggers of asthma exacerbations include air pollution, cigarette smoking, allergens and respiratory tract infection (RTI) with virus and bacteria (1). Interactions between these triggers likely occur, with the underlying host susceptibility also playing a role in exacerbations, which renders it challenging to ascertain the exact mechanisms and interplay involved in exacerbations and develop effective therapeutics. Despite the importance of all of these triggers in asthma pathogenesis, in this review we focus on the role of bacteria and viruses and their crosstalk, with the role of other environmental factors recently reviewed elsewhere (31, 32).

**Figure 2:**
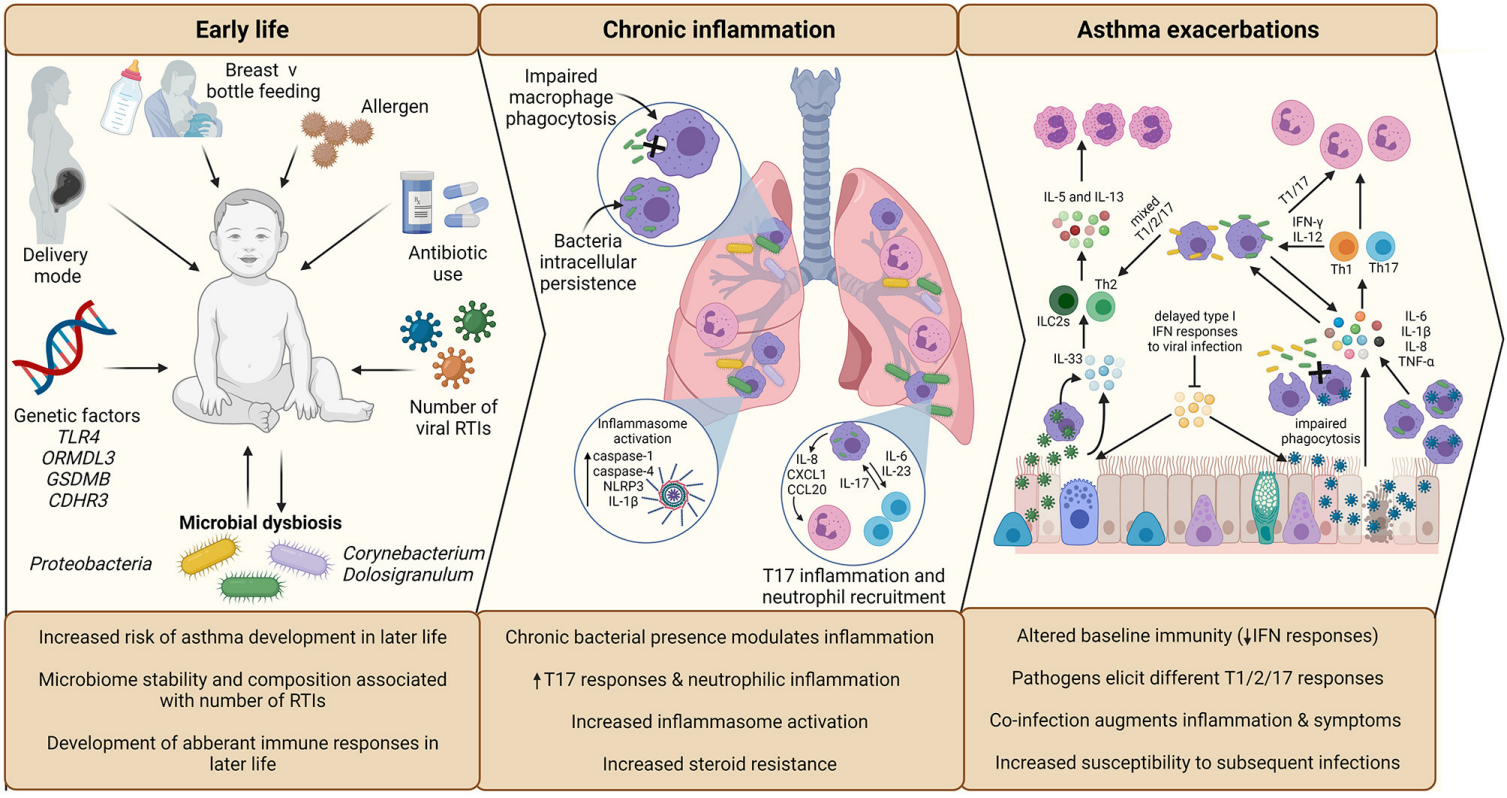
Overview of the impact of infection in early life asthma development, on chronic inflammation in established asthma and during asthma exacerbations. The development and stability of the microbiome in early life is associated with allergen exposure, mode of delivery, feeding method, and antibiotic use. The composition of the microbiome is associated with the number of RTIs during infancy, which is subsequently associated with increased risk of asthma development in later life. Microbial dysbiosis resulting in microbiome profiles enriched in Proteobacteria particularly is associated with development of asthma and aberrant immune responses in later life. During established asthma, chronic colonization of the airway by Proteobacteria is associated with modulation of airway inflammation. NTHi persistent airway colonization causes inflammatory responses switching to T17, neutrophilic inflammation, increased inflammasome activation, which is linked to increased steroid-resistance. As a possible consequence of chronic bacterial colonization, baseline immunity in asthma is altered, including decreased antiviral immunity (IFN responses) and macrophage function (phagocytic ability). Viral infection is established due to delayed antiviral responses, which causes increased asthma symptoms, resulting in a virally driven exacerbation. Different viruses cause different disease pathology (e.g., increased cellular cytotoxicity following IAV infection compared to RV) and induction of T2-low (IAV) or T2-high (RV) responses. Bacterial infection also causes exacerbations and are likely contributors to exacerbation symptoms following viral infection, as bacterial outgrowth occurs due to impaired phagocytosis and macrophage immune response sensitization induced by viral infection. Again, different bacteria induce different responses, with Mcat reported to induce a mixed T1/2/17 response, whereas NTHi drives a T1/T17 pro-inflammatory response. Co-infection can augment inflammation and asthma symptoms, which may impact on treatment failure/success during exacerbation. Created using BioRender.com.

Respiratory tract viral infections are also linked to early life asthma development. The advent of non-culture-based methods of bacterial detection, such as 16S rRNA sequencing, has increased appreciation for the role of potentially pathogenic bacteria in asthma development and pathogenesis (Figure 2). Airway colonization by potentially pathogenic bacteria within 1 month of life is suggested to predispose individuals to asthma (33), indicating that modulation of airway immunity may occur even prior to respiratory tract viral infections. Indeed, bacteria-virus co-infection of the airway results in more severe exacerbations and hospital readmission of individuals with chronic respiratory disease (34). These observations suggest an underappreciated crosstalk is occurring during bacteria-virus co-infections in the airway.

Here we will review the currently understood role of infection in three aspects of asthma: (i) asthma exacerbations, (ii) early life development of asthma, and (iii) established asthma and inflammatory phenotypes. We will discuss the emerging evidence of crosstalk between bacteria and viruses and the host, with a particular focus on how co-occurrence of pathogens may impact asthma pathogenesis and inflammatory phenotypes. Finally, we will discuss how our increasing appreciation for the co-occurrence of certain respiratory pathogens and their crosstalk may reveal novel treatment modalities to improve outcomes for individuals with asthma.

## Asthma Exacerbations: The Role of Infection

The role of viral infection in asthma exacerbations has been known for decades, with early studies showing associations between respiratory pathogens and asthma attacks (35), with experimental rhinovirus (RV) inoculation of volunteers inducing airway hyperresponsiveness providing evidence for viral infection contributing to exacerbations (36, 37). A systematic review by Papadopoulos et al., found 9 viruses associated with exacerbations of asthma, with virus prevalence differing between adults, children (6–17 years old) and infants (<6 years old) (38). RV is the most common virus detected during an exacerbation in both children (55%) and adults (29%) (38), with detection of RV up to 5 days prior, being found to be significantly associated with the development of an exacerbation (39). However, the exact causative role of viral infection in asthma exacerbations remains unclear, with other studies demonstrating no changes in asthma symptoms following viral challenge (40, 41).

Furthermore, the development of culture-independent methods has advanced our ability to detect respiratory pathogens that are difficult to culture, resulting in an expansion of our knowledge of the pathogens present in the airways and associated with exacerbations. For example, the presence of RV-C was likely previously overlooked due to difficulties in growing and culturing RV-C *in vitro* (42), but is now suggested to be the RV strain associated with more severe illness (43). However, the majority of patients presenting with asthma exacerbations are not screened using multiplex PCR viral detection systems meaning that the true association of different viral pathogens with asthma exacerbations is difficult to interpret. Numerous bacterial pathogens, other than the original atypical bacterial pathogens *Mycoplasma pneumoniae* and *Chlamydia pneumoniae* (44), are now implicated in asthma, including *Haemophilus influenzae, Moraxella catarrhalis* (Mcat), *Streptococcus pneumoniae, Haemophilus parainfluenzae, Klebsiella pneumonia*, and *Bordetella pertussis* (45–47). Studies have consistently identified enrichment of Proteobacteria in asthma (48) and have shown *H. influenzae* and Mcat to be associated with clinical outcomes. *H. influenzae* is associated with poor asthma control (49), steroid-resistance (45, 50), neutrophilic inflammation (45, 51, 52), and increased disease severity (16, 49). In contrast, Mcat is associated with the induction of mixed inflammatory phenotypes (45, 51, 53), loss of asthma control in children (54), and increased number of exacerbations in children (55). As such, this review will mainly focus on these two clinically important Proteobacteria, as well as three viruses of interest: RV, influenza, and respiratory syncytial virus (RSV).

### Mechanisms Contributing to Infection-Induced Asthma Exacerbations

**Figure 3:**
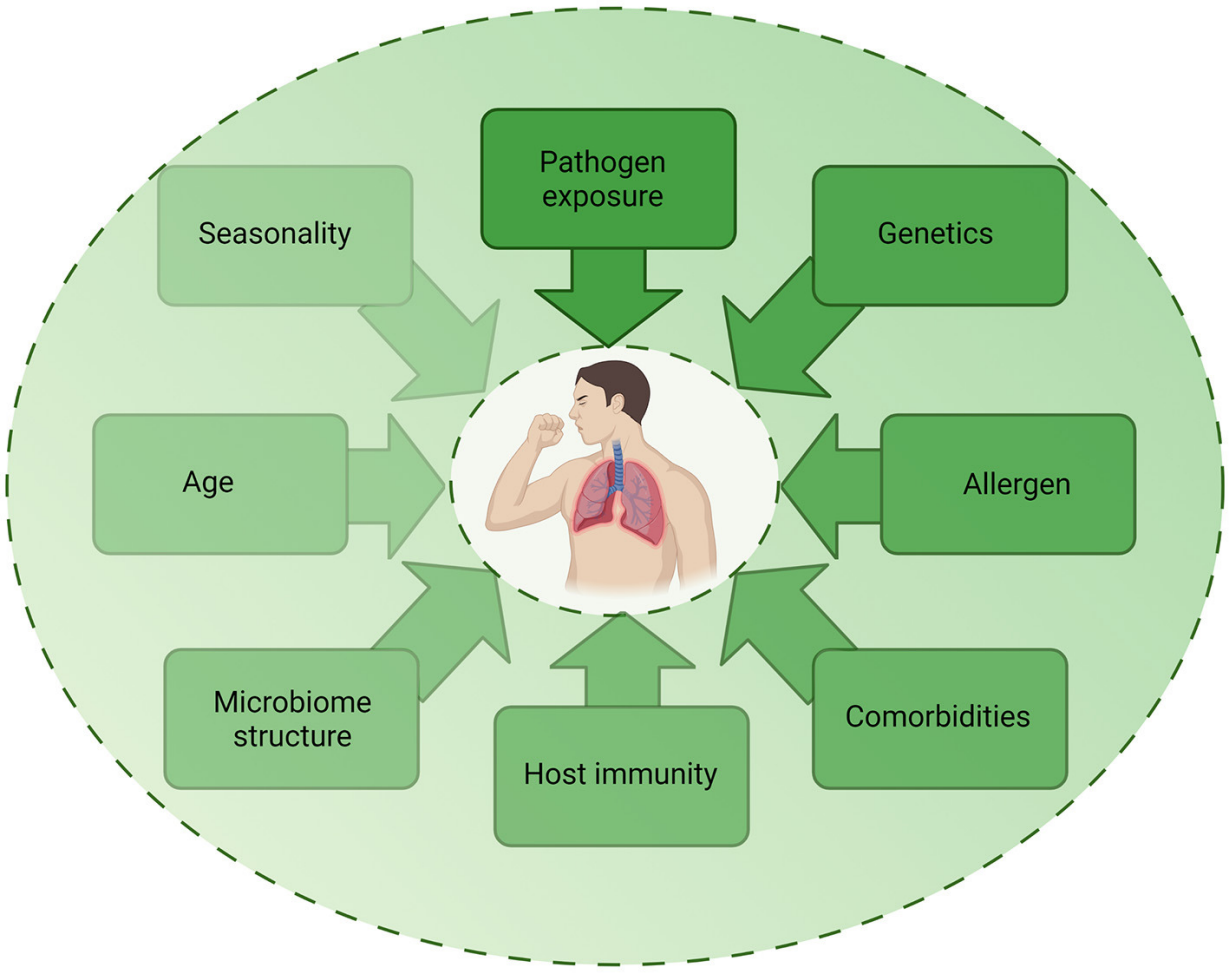
Factors contributing to infection-induced exacerbations. The role of infection in exacerbations are likely to be multifactorial, with several host, pathogen, and environmental factors impacting on the contribution of infection to an exacerbation. Each factor may be multi-directional and directly or indirectly impact on another factor, highlighting the complex mechanisms involved in exacerbations. Created using BioRender.com.

Despite epidemiological studies demonstrating associations between infection and asthma exacerbations, the exact mechanisms by which these different pathogenic triggers drive exacerbations remain unclear. As detection of pathogens during exacerbation do not prove causation, it is important to consider how other factors may contribute to exacerbations (Figure 3). The multifactorial nature of asthma is such that the underlying atopic status, timing of allergen exposure or asthma severity may influence the host response to infection and account for differences observed in experimental challenge or observational studies. For example, influenza infection causes increased epithelial cell lysis compared to RV infection (21), but the presence of an atopic environment was shown to increase RV cytotoxicity (56) and be protective against influenza A infection (57, 58). The timing of allergen exposure and viral challenge also appears to influence outcomes. Sensitization and exposure to high allergen levels followed by viral exposure increases the risk of hospital admission (59), whereas individuals challenged with allergen after RV infection demonstrated higher levels of allergic responses compared to non-allergic individuals challenged with RV (60). As such, differences in clinical or experimental study outcomes may be due to a number of host or pathogen factors including genetics, allergen sensitization, asthma endotype, comorbidities, age, experimental timing and dosing, seasonality, and viral strain. Nonetheless, a study using omalizumab, an anti-IgE monoclonal antibody, was found to reduce exacerbations even during seasonal peaks (61), providing evidence for allergen-viral interactions in exacerbations.

**Figure 4:**
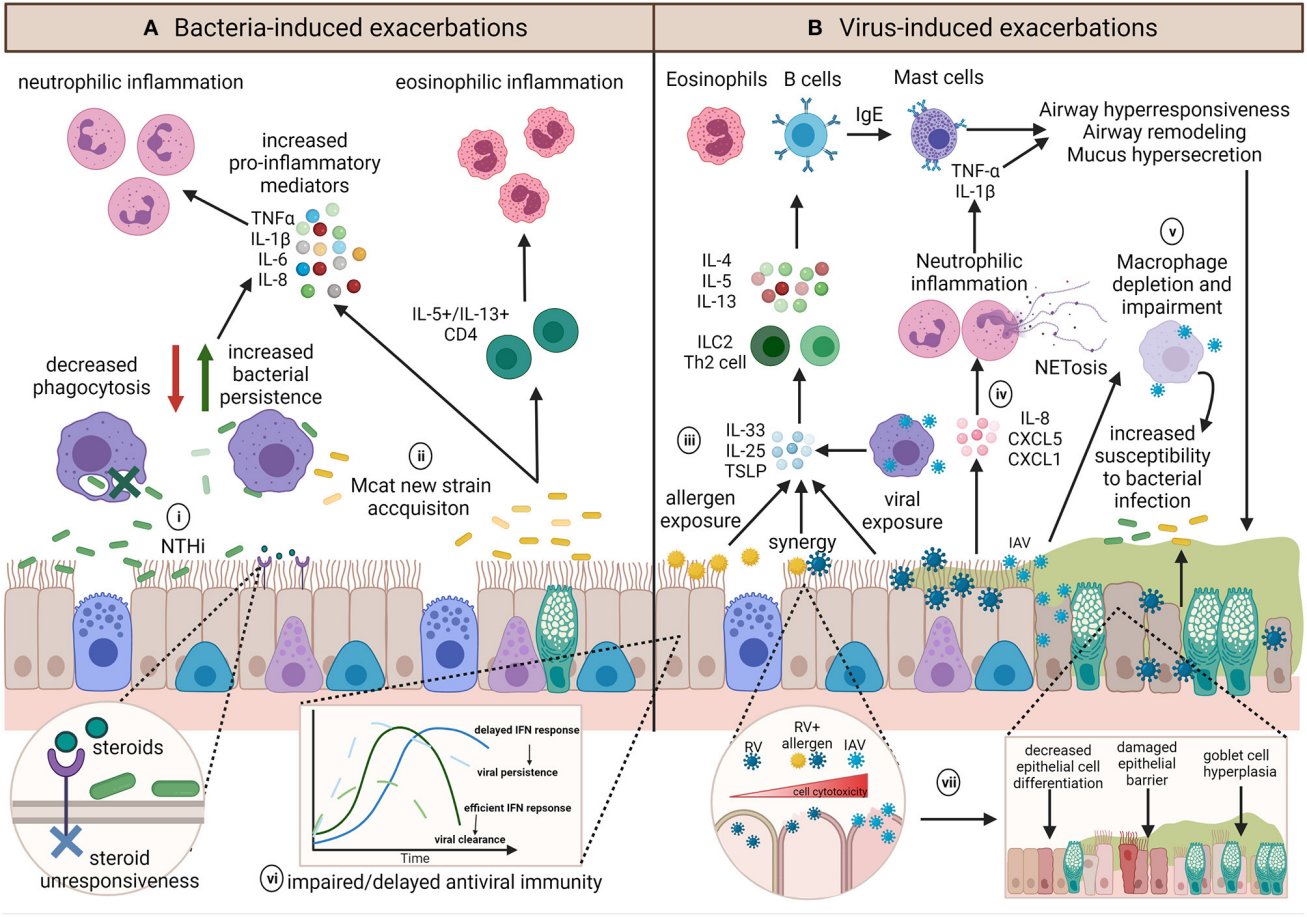
Summary of the diverse potential mechanisms contributing to and influencing infection-induced exacerbations. **(A)** The role of bacteria in exacerbations are not well-known but inflammatory profiles associated with exacerbations appear to be pathogen dependent. (i) NTHi infection results in upregulation of pro-inflammatory mediators and is associated with persistent infection, neutrophilic inflammation and steroid-resistance. Chronic NTHi airway colonization may occur as a result of macrophage impairment in phagocytosis. (ii) On the other hand, exacerbations with Mcat are associated with acquisition of a new Mcat strain and is associated with both neutrophilic and eosinophilic inflammation. **(B)** Viral induced exacerbations can induce the hallmarks of asthma exacerbations through upregulation of T2 responses. (iii) Epithelial cells and macrophages contribute to IL-33, IL-25, and TSLP release, resulting in ILC2 or Th2 release of IL-4, IL-5, and IL-13 to upregulate eosinophils and B cells, which produce IgE and subsequently activate mast cells, all of which induce airway hyperresponsiveness, remodeling, and mucus hypersecretion. (iv) Conversely, some viruses have been shown to induce IL-8, CXCL5, and CXCL1 release, resulting in neutrophilic inflammation and epithelial damage *via* neutrophil production of NETs. (v) Influenza infection can impact on macrophage function or result in macrophage depletion, which along with mucus plugging, and may render the airway susceptible to persistent bacterial infection, and may further act to exacerbate airway inflammation. Underlying host factors such as (vi) impaired host antiviral immunity may result in an altered baseline immune profile that contributes to a dysregulated host response, whereas (vii) allergen sensitization or exposure may synergistically augment inflammation and increase cell lysis and damage during viral infection. Created using BioRender.com.

Different respiratory pathogens, even different strains of the same pathogen, induce distinct responses (62–64). However, the underlying inflammatory environment and host immune responses may further influence infection outcomes (Figure 4). Investigations building on experimental challenge studies have identified a number of potential host impairments which may contribute to infection-induced exacerbations. These include ciliary dysfunction, ciliostasis, mucus hypersecretion (65), goblet cell hyperplasia (66–70), altered and dysregulated baseline inflammatory gene expression (71), impaired antiviral responses (72), delayed interferon responses (73), decreased levels of the innate immune surfactant protein (SP)-D (74), altered macrophage phenotype (75), and decreased macrophage phagocytosis (76, 77). The exact mechanisms of how each individual pathogen contributes to an asthma exacerbation is multifactorial and is likely dependent on pathogen strain/subtype and a myriad of host factors. These factors become even more complicated when we consider the potential of bacteria-virus interactions with the host and how these may influence the development of exacerbations. We will next consider how bacteria-virus crosstalk during co-infection may precipitate asthma exacerbations.

### Double Trouble? The Role of Co-infections in Asthma Exacerbations

Bacterial and viral airway co-infections in chronic respiratory disease are an important clinical consideration due to individuals suffering from more severe illness, increased exacerbation risk and increased hospital readmissions (34, 78, 79). *H. influenzae* in particular has been found to be co-detected with the three main etiological viral agents of asthma exacerbations, influenza, RV, and RSV, resulting in increased disease severity and likelihood of viral infection when *H. influenzae* is present (46, 55, 79–83). Notably, associations between *H. influenzae* and RV in the airway are not limited to asthma, with longitudinal studies in COPD demonstrating the presence of both NTHi and RV associating with decreased lung function, increased exacerbation risk and increased airway inflammation (84, 85).

In contrast, Kloepfer et al. analyzed the impact of RV-bacteria co-infection in children and found that *H. influenzae* did not associate with increased respiratory symptoms. Instead, carriage of Mcat was more significantly associated with increased symptoms and asthma exacerbations, with no associations between pathogen and atopic status (78). This association between Mcat and RV in asthmatic children may be due to similarities in the timing of RV and Mcat circulation, as well as age-dependent host factors. As the seasonal pattern of Mcat coincides with RV, it is possible that RV-Mcat or RV-host interactions may promote Mcat pathogenesis in children.

#### Modulation of Host Cell Surfaces

Several studies have attempted to elucidate the mechanisms underlying bacteria-virus crosstalk during co-infection using various combinations of model systems, respiratory pathogens, and infection protocols. Modulation of host cellular surfaces by pathogens may increase the ability of other pathogens to adhere to airway cells and establish infection (86). *In vitro* studies have shown that the main cellular receptor for RV, ICAM-1, is upregulated following infection of epithelial cells and monocytes by NTHi (87) and Mcat (88) and is also utilized by NTHi for adherence and invasion (89). Increased RV infection following pre-incubation of epithelial cells with NTHi, was associated with NTHi-mediated upregulation of ICAM-1 (90).

Similarly, both IAV and NTHi exploit the presence of sialic acids present on host cells to facilitate attachment and entry into host cells (91–94). IAV strains preferentially bind to α2,6-linked sialic acid residues (91), whilst NTHi can bind to α2,6- or α2,3-linked sialic acid residues depending on the differential expression of NTHi outer membrane proteins HMW1/2 or Hia (92–96). An intriguing concept is that the use of sialic acid by both NTHi and IAV to establish infection could in fact predispose the airway to infection by other respiratory tract pathogens such as *S. pneumoniae*, which can co-infect the airway with NTHi (97). Work using *in vitro* and *in vivo* infection models demonstrated that desialylation of epithelial cells following IAV infection resulted in increased *S. pneumoniae* adhesion to host cells (98).

#### Modulation of Host Immune Responses

As well as influencing pathogen adherence to the respiratory tract, co-infection also modulates host responses. Viral infection can result in a phenomenon termed immune paralysis, characterized by immunological defects such as decreased macrophage phagocytosis (99). Murine alveolar macrophages (AM) display reduced phagocytosis in response to secondary bacterial challenge a week after initial influenza virus challenge, resulting in higher bacterial burden (100). Another study reported sustained desensitization of AM lasting for several months following influenza infection, resulting in reduced chemokine production and NF-κB activation, leading to reduced airway immune cell recruitment during secondary bacterial challenge (101). Furthermore, influenza infection of mice reduced the number of AM, increasing susceptibility to secondary *S. pneumoniae* infection (102). These impacts are not limited to influenza: RV infection of human AM also resulted in impaired pro-inflammatory cytokine release and modulation of phagocytosis capacity during subsequent AM challenge with LPS or *E. coli* bioparticles (103).

In contrast, studies have demonstrated augmented pro-inflammatory responses during co-infection, which may translate to the enhanced inflammation, increased illness severity and hospital admissions observed during asthma exacerbations (34, 78). Co-infection of epithelial cells with RV and NTHi increased release of neutrophil chemoattractants, CCL20 and CXCL8/IL-8, compared to infection with either pathogen alone (104). Frick et al. found that co-infection with NTHi and RV resulted in increased neutrophil adherence *in vitro* and leukocyte recruitment in an airway infection murine model (87). Similarly, neutrophil infiltration was observed in a NTHi-IAV murine co-infection model, which also demonstrated increased levels of pro-inflammatory cytokines (105). Conversely, a recent study utilizing an allergic airways disease murine model reported that the microbiome structure was influenced by the allergic inflammatory environment, which in turn reduced the impact of influenza–*S. pneumoniae* co-infection (106). Consideration of disease setting and other potential underlying host factors is important; not only does the microbiome appear to influence responses to infection, but murine models of allergic airways disease have shown the presence of an atopic environment also influences infection outcomes (58).

#### Does the Timing of the Immune Response During Co-infection Influence Outcomes?

Although bacteria-virus interactions have historically been investigated in the context of secondary bacterial infections succeeding viral infection, our increasing knowledge of airway microbiota presence requires careful consideration of host-pathogen dynamics. Invasion and persistence within epithelial and immune cells enhances survival of both NTHi and Mcat (107–115), with airway persistence varying from days to years (85, 116–119). This chronic airway presence may modulate host immune responses and influence the progression of a subsequent viral infection. A number of *in vitro* studies have reported modulation of anti-viral responses by bacteria. Heinrich et al. found that, *in vitro*, Mcat-mediated TLR3 downregulation led to decreased secretion of interferons such as IFN-β and IFN-λ, resulting in increased susceptibility of epithelial cells to a subsequent RV infection (120). In contrast, NTHi infection upregulated TLR3 receptor expression in airway epithelial cells prior to RV infection. However, as NTHi also upregulated ICAM-1 expression, levels of RV attachment to cells increased, resulting in synergistic IL-8 release (121). In a separate study, NTHi infection also enhanced epithelial cell TLR7 expression and type I IFN responses. However, this study did not assess whether this modulation of anti-viral immunity influenced a subsequent viral response (122). Importantly, TLR7 expression by AMs from severe asthma is reduced (123). As such, NTHi-mediated upregulation of type I IFN responses *via* TLR7 may not be recapitulated in the severe asthmatic airway.

Unraveling host-pathogen interactions outside of the lung environment is complex and challenging, highlighting the importance of translating *in vitro* and murine *in vivo* studies into human disease settings. Human challenge models may help us better reconcile observations and associations from clinical cohort studies and experimental models. For example, a recent eloquent study by Habibi et al., identified an association between a neutrophil transcriptomic signature with increased susceptibility for RSV symptomatic infection using experimental RSV nasal inoculation of healthy volunteers (124). Thus, the presence of bacteria such as NTHi, which is associated with a neutrophilic phenotype, may predispose individuals to symptomatic viral infection, which may consequently result in development of an asthma exacerbation. Indeed, a study by De Steenhuijsen Piters et al., found that children with *H. influenzae*-dominant microbiomes were more likely to be hospitalized with RSV-induced bronchiolitis and was associated with neutrophil and macrophage transcriptomic signatures (125).

However, asthma is heterogeneous and caution must be used when interpreting results from single clinical studies in humans. Various differences between study cohorts may impact on conclusions, including factors such as age, underlying inflammatory phenotype, current treatment, and microbiome structure. For example, varying clinical cut offs for eosinophilic and neutrophilic inflammation (126, 127), as well as the use of molecular markers of T2 inflammation (*CLCA1, SERPINB2*, and *POSTN*) (53) can be used to stratify patients. The diverse nature of asthma patients may account for the conflicting evidence of bacterial abundance and diversity in asthma (128–131), with some studies also indicating that microbiome diversity and abundance differs with T2-low/high or eosinophilic/neutrophilic inflammatory phenotypes (49, 51, 53), and steroid-responsiveness (128, 131). As such, it is important to consider how the aforementioned factors may collectively impact on study outcomes when considering the role of bacteria in asthma.

Overall, the impact of bacterial pre-exposure on host immunity and subsequent viral infection appear conflicting; the differences in the results of these studies may be due to different infection models, pathogen strains/combinations and infection protocols used. It is important to determine whether bacterial presence in the airway is a protective, causative, or contributing agent for exacerbations, or a biomarker for exacerbation risk, depending on underlying host factors. However, whilst the role of RTI in causing asthma exacerbations has been known for at least 30 years, there is now emerging evidence that RTI may also contribute to asthma development. This emerging evidence is the focus of the next section.

## Early Life: The Role of Infection

Asthma is regarded as a hereditary trait and the use of genome-wide linkage studies of twins and families have resulted in estimates of heritability that range from 35 to 70% (132–134). Numerous candidate genes have been suggested to be associated with asthma, with Genome Wide Association Studies (GWAS) identifying asthma susceptibility loci (135, 136). However, independent GWAS do not completely overlap in their findings, likely due to differences in environmental exposures of individuals within study cohorts (137). Environmental factors play a considerable role in asthma development (Figure 2), with Thomsen et al. identifying that environmental factors explained a higher proportion of the variation in age of onset of asthma (66%) compared to genetic factors alone (34%) (133). Earlier GWAS and candidate gene association studies did not include the possibility of environmental factors interacting with host genetics, which may explain the lack of overlap between studies (137). Building on candidate gene associations and GWAS, gene-environment interaction studies may unveil novel genes that are only significant for asthma susceptibility when combined with the appropriate environmental exposure (138). Such environmental factors include smoking, pollution, mold, farming-related exposures, dust, and respiratory tract infections (134). This section will discuss the potential role of respiratory tract infections in predisposing individuals to asthma development.

### Gene-Bacteria-Virus Interactions Influencing Host Immunity in Early Life

Given that asthma is characterized by dysregulated and exacerbated immune responses, as a result of genetic-epigenetic-environmental interactions, assessing the influence of infection on host immunity has been an important area of investigation. It has been suggested that viral infection in combination with genetic predisposition increases the risk for childhood asthma development. A GWAS identified the first known asthma susceptibility locus, 17q21 (139), which is associated with childhood respiratory infection (140). Notably, investigation of this locus in two cohorts—the Childhood Origins of Asthma (COAST) birth cohort and the Copenhagen Prospective Study on Asthma in Childhood (COPSAC) birth cohort—found that responses to RV, but not RSV, were associated with 17q21; RV-stimulated PBMCs expressed higher levels of two 17q21 genes, *GSDMB* and *ORMDL3* (141). Subsequent *in vitro* investigations silencing *ORMDL3* reported attenuated pro-inflammatory responses and reduced *ICAM1* expression, providing a potential mechanistic basis for *ORMDL3* and RV susceptibility in asthma (142). Given that ICAM-1 also serves as a receptor for NTHi and is upregulated in response to NTHi presence (90), it is possible a complex interplay occurs between genetic susceptibility, chronic NTHi airway colonization and RV infection. Furthermore, recent work has identified a potential prominent role of RV-C in childhood asthma development. The RV-C entry receptor is CDHR3, the product of *CDHR3*, which was identified by Bønnelykke et al. in a GWAS to be an asthma susceptibility gene (143). The *CDHR3* gene is associated with increased RV-C detection and risk of respiratory tract illness (144), further highlighting the importance of gene-environment interactions wherein viral infection is a critical environmental exposure.

Genetic alterations may also predispose individuals to recurrent bacterial infections. The Toll Like Receptor (TLR)4 polymorphism, Asp299Gly, is associated with increased gram negative bacterial infections (145), lower cell surface TLR4 expression, and impaired TLR4-mediated lipopolysaccharide (LPS) signaling (146). Individuals carrying the Asp299Gly TLR4 polymorphism that were colonized with Mcat at 2 months or *H. influenzae* at 13 months of age were at an increased risk of asthma development (147). Stimulation of PBMCs *ex vivo* found that this particular polymorphism results in decreased LPS-induced IL-10 and IL-12 responses and was independently associated with atopic asthma (148). However, an earlier study did not find any associations between TLR4 polymorphisms and asthma (149), likely due to potential differences in cohort-specific gene-environment interactions. Indeed, Terasjarvi et al. did not find an association between TLR4 polymorphism and asthma risk alone, but instead between TLR4 polymorphism, *H. influenzae* colonization, and asthma risk (147), highlighting the need to consider multiple host-environment factors to unravel the complex and multifaceted nature of asthma.

### Viral Infection

Early studies initially identified viral infection to be significantly predictive of asthma development, resulting in a focus on early life viral infections and asthma development (150–152). In infancy, wheezing is one the earlier predictors of asthma, heavily implicating viruses such as RV and RSV. Early work reported RV-associated wheezing during infancy was the strongest predictor for wheezing in the third year of life (151) and more severe RV infections resulting in hospitalizations during infancy were associated with asthma development (150). Retrospective studies have identified children with early life RSV infection, particularly those requiring hospitalization, to be more at risk of asthma development (153). Children who were hospitalized as a result of severe RSV bronchiolitis were shown to be at risk of developing asthma by age 18 (154). Indeed, in this Swedish study, 30% of children who developed severe bronchiolitis developed reactive airways disease by age 7 compared to just 3% of controls. Studies in animal models have shown that RSV infection can upregulate IL-4, IL-5, IL-13, and the T2 chemokine CCL17 (155). This increase in T2 cytokines was postulated by the authors to be a result of NK cell depletion and subsequent reduction of IFNγ expression by RSV. This RSV-induced switching to a T2-phenotype may explain some of this susceptibility to asthma development. Furthermore, *in vitro* work has shown decreased RSV load was associated with the epigenetic regulation of RIG-I following IFNγ priming (156). Thus, the timely development and maturation of immune responses may depend on early life pathogen exposure.

Synergistic interactions between virus and allergen exposure may also contribute to asthma development. RV or RSV induced illness in infancy is associated with subsequent wheezing, but was found to be most significantly associated in children who were sensitized when <2 years old (157). The importance of age and timing of allergen sensitization was confirmed in a separate cohort, with 65% of children sensitized by 1 year of age identified to have asthma by 13 years of age, compared to only 17% of children sensitized by 5 years of age (158). A synergistic effect of allergen exposure and viral infection was proposed following inoculation of volunteers with RV and allergen challenge, which augmented allergic airway responses compared with RV inoculation alone (60). Subsequent studies confirmed this observation, with increased risk of hospitalization associated with allergen sensitization and viral infection during exacerbations of childhood asthma (59, 159). The mechanisms underlying how virus-allergen exposure synergistically contributes to asthma development are not understood, however a murine model of RSV infection and allergen sensitization found that recurrent RSV infections of sensitized mice resulted in T2 cytokine production, increased serum IgE and airway hyperresponsiveness (160).

Although viral infections are thought to be transient and provoke an acute inflammatory response, associations between viral infection and asthma development have prompted investigations into how viruses cause chronic inflammation. Studies have identified that, following the initial viral insult, trace amounts of virus are detectable in the lung and are associated with increased eosinophilic inflammation, airflow obstruction and lower FEV_1_ (161). A murine model of chronic respiratory disease demonstrated this persistent inflammation and airway hyperresponsiveness was mediated by lung macrophages activating invariant natural killer T (iNKT) cells to release IL-13, a finding that, importantly, was also observed in a human cohort (162). Thus, viral infection in early life may initiate immunological changes that persist and precipitate asthma development.

As most children are exposed to viruses during early life, but not all children go on to develop asthma, it is likely that other co-factors are involved. Intriguingly, analysis from the COPSAC cohort found that no specific infectious trigger, but rather, the number of respiratory episodes predisposes individuals to asthma in later life (163). Studies have shown that in fact, enrichment of certain bacteria in the respiratory tract during early life is associated with recurrent RTIs and increased risk of asthma development.

### The Role of Microbial Dysbiosis in Asthma Development

Our understanding of the role of bacteria in the airway is only recently being advanced due to the incorrect dogma of lung sterility, which has prevailed since the late nineteenth century. As the study of the lung microbiome is a relatively young field, it faces technical and methodological challenges, particularly given the low microbial biomass of the lung environment (164). Despite this, common lung microbiome profiles have been successfully identified, allowing for comparisons of the lung microbiome between health and disease (48, 164, 165). The dynamics of early life microbiome development are suggested to influence the maturation of host immune responses and consequently respiratory health, with childbirth delivery mode and breastfeeding associated with microbiome structure, development, and temporal stability (166–169).

One theory that attempts to explain the increased incidence of asthma during the twentieth century is the “hygiene hypothesis” (170, 171). This theory, backed by a body of evidence, postulates that modern day cleanliness and sterile environments have promoted the development of allergic diseases, such as asthma, by reducing the exposure of individuals to non-infectious organisms during childhood (172–176). Accumulating evidence indicates that there is a timely window of opportunity during early life wherein disruptions in microbial colonization can increase the risk of asthma development in later life. Indeed, Bisgaard et al. reported that children who were colonized within 1 month of life by potentially pathogenic bacteria, including *H. influenzae* and Mcat were more likely to develop asthma by the age of 5 (165). Building on this seminal work, further analysis found associations between microbiome stability and respiratory health in the first 2 years of life (169). More stable microbiota profiles were dominated by *Moraxella* and were associated with a lower number of consecutive respiratory infections (169). In contrast, other studies have found *Moraxella*-dominant microbiomes were associated with younger age of first upper RTI (177), increased number of RTIs (178), and increased severity of illness (177). The timing of *Moraxella* colonization appears to be crucial. In line with the original findings of Bosch et al., *Moraxella* becomes the dominant microbiome community member in healthy children much later (2–3 months) (178). Premature microbiome maturation involving an early transition to a *Moraxella-*dominant microbiome profile is associated with an increased number of RTIs (178).

Conversely, *Haemophilus-*dominated microbiome profiles are less temporally stable (169, 177), and emerge later than *Moraxella* (178). Characterization of the nasopharyngeal microbiome in the first 5 years of life found that the presence of *Haemophilus* was associated with increased respiratory tract illness symptoms, both synergistically and independently of viral presence (RSV and RV), indicating a bacteria-dependent contribution to respiratory illness (179). Furthermore, *H. influenzae* colonization was more significant in an “infection and allergy prone” subgroup of children in a retrospective analysis investigating the relationship between bacterial colonization and respiratory illness in children between 6 months and 5 years of age (180). This study also found a significant association between *H. influenzae* and influenza infection, which was not observed for other respiratory bacteria. It is not clear whether *Haemophilus* associations with early life asthma development is a consequence of altered microbiome maturation and immune training prior to the establishment of a *Haemophilus-*dominant microbiome. Overall, the mechanistic influence of colonizing bacteria on host responses prior to and during viral infection is not well-understood, highlighting the need to also characterize the functional relevance of early life bacterial colonization on immune tone, rather than just drawing correlations between bacterial presence with respiratory health outcomes.

Nonetheless, evidence indicates that airway bacteria modulate host immune responses. Exaggerated responses were observed in PBMC isolated from children prior to asthma development in later life, including increased IL-5, IL-13, IL-17, and IL-10 when exposed to *H. influenzae* and Mcat (181). Building on these observations, murine models of airway disease demonstrate a potential functional consequence of airway colonization by potentially pathogenic bacteria. Mice colonized with NTHi after only 3 days of life exhibited an exacerbated response when later challenged with an allergen, implicating NTHi in aberrant host immune development (182). Similarly, Mcat and allergen exposure synergistically resulted in an IL-17-mediated exacerbated immune response in mice (183). Development of aberrant host immune responses following early life colonization by bacteria may influence host responses to infections and contribute to asthma development. For example, mucosal-associated invariant T (MAIT) cells are important antibacterial innate-like immune cells which depend on microbiota-derived signals for timely development and maturation (184, 185). Thus, as a healthy microbiome appears to be crucial for MAIT cell-mediated resistance to infection, dysbiosis of the airway microbiome by pathogens such as NTHi and Mcat in early life may influence MAIT cell development. Indeed, MAIT cells are activated upon NTHi infection of macrophages *in vitro* (186) and are important for host resistance to other respiratory pathogens (187, 188). Thus, dysregulation of MAIT cell function may contribute to aberrant immune responses in asthma.

As evidence indicates that these bacteria also chronically colonize the airway of individuals with established asthma, we will next explore how these bacteria can contribute to the development and persistence of asthma inflammatory phenotypes and modulate airway immune responses.

## Established Asthma: The Role of Colonizing Bacteria in Shaping Host Immune Responses and Inflammatory Phenotypes

### Host-Pathogen Crosstalk During Persistent Airway Colonization

Although the importance of lung microbiome composition differences between health and disease is becoming apparent, the complexities of host-microbiome interactions are only now beginning to be appreciated (189). Increasing evidence indicates that the gut microbiome modulates the host mucosal defense response. However, less is known about the role of the lung microbiome in regulating the host immune response (190). Human lung microbiome studies have mainly consisted of using metagenomics on large cohorts to identify the microbiome composition. Although metagenomics is a powerful tool, measuring the relative abundance of bacterial species does not necessarily correlate with the activity of the microbes present (191).

As such, studies are now beginning to correlate microbial activity with host gene expression. Using a combination of shotgun RNA sequencing for microbial identification and host differential gene expression analysis, Castro-Nallar et al., identified a specific host gene profile associated with the presence of Proteobacteria (192). Expanding on this work, Perez-Losada et al., used dual transcriptomic profiling to assess differences in the host and microbial functional properties of asthmatic children and healthy controls (193). They found differences in bacterial metabolism-associated genes between the metatranscriptomes of asthmatic and non-asthmatic individuals, with host *IL1A* expression associated with bacterial adhesion (193). Further metabolic differences in the asthmatic bronchial microbiome was observed by Durack et al., who found enrichment of bacterial short chain fatty acid (SCFA) and amino acid metabolism in atopic asthmatics (53). Interestingly, this study also found functional differences in the microbiome were associated with ICS responsiveness, suggesting members of the microbiome may influence treatment efficacy. Unfortunately, due to the experimental limitation of low bacterial biomass in the lung, the sample size was too small to perform meaningful comparisons between T2 low/high individuals. Nonetheless, such studies demonstrate the importance of using metatranscriptomic approaches to determine the functional impact of these altered microbiome profiles in the development and progression of asthma and modulation of host immune responses during stable periods of disease.

### NTHi and the Neutrophil Inflammatory Phenotype

Numerous studies have identified associations between Proteobacteria and some of the main clinical features of asthma including increased bronchial hyperresponsiveness (129) and airway inflammation (52, 129), such as a mixed T1/T2/T17 inflammatory response (16, 194). Expansion of Proteobacteria appears to be inversely correlated with eosinophil levels but is significantly associated with increased neutrophil levels (16, 45). In particular, NTHi is detectable during stable periods of disease and is associated with T2-low inflammation, increased neutrophils and steroid-resistance [Figure 4; (45, 49)]. Development of steroid-resistance in asthma has been linked to increased NLRP3, caspase-1, and IL-1β responses to NTHi infection (50, 195). Experimental models trying to reconcile the observations of NTHi and neutrophilic inflammation in cohort studies have shown that suppression of IL-1β-mediated responses prevented the development of the steroid-resistant features of asthma (50). However, it remains unclear whether NTHi contributes to the progression of severe, neutrophilic, steroid-resistant asthma, or if NTHi takes advantage of the chronically inflamed and damaged airway that is characteristic of severe asthma to colonize the lung.

To try and untangle the complex mechanisms underlying this question, murine studies have investigated the development of neutrophilic disease following NTHi colonization. NTHi infection of ovalbumin (OVA)-sensitized mice resulted in an influx of IL-17+ macrophages, neutrophils, and lymphocytes, with eosinophilic inflammation reduced (196). Despite an influx of immune cells, NTHi infection was sustained in mice with allergic airways disease compared to non-allergic mice (197). The combination of allergic airways disease and infection contributed to the emergence of a steroid-resistant neutrophilic phenotype, suggesting the synergy between prior host allergic sensitization and subsequent NTHi infection promotes disease development. However, the duration of these aforementioned murine studies may be too short to ascertain whether the neutrophilic phenotype persists or if the neutrophilic inflammation was a transient event with inflammatory resolution occurring after the chosen study endpoint. Yang et al., extended the experimental endpoint to 2 months using OVA-sensitized mice and repeated low dose infections with NTHi to model NTHi chronic colonization of the airway (198). As a consequence, by the 2-month (56 day) endpoint, the inflammatory profile switched from T2-associated eosinophilic inflammation to T17-associated neutrophilic inflammation, accompanied by Treg immunosuppression and impaired macrophage phagocytosis. Together, these murine studies suggest that the combination of allergic airways disease and NTHi infection drives the neutrophilic inflammation, with this inflammatory phenotype still present 2 months after the first NTHi exposure. Thus, minimizing the burden of chronic NTHi presence in asthma could reduce the development of steroid-resistance and improve outcomes for patients requiring steroids to manage their symptoms.

## Changing the Trajectory: Strategies to Reduce the Burden of Pathogens in the Airway

As patients with T2-low asthma tend to respond poorly to current therapies, identifying which components of T2-low inflammation to therapeutically target is of utmost importance. However, current therapies targeting these alternate pathways have not shown to be effective. The IL-17 family of cytokines is implicated in more severe and neutrophilic forms of asthma, but a clinical trial treating patients with brodalumab, a monoclonal antibody targeting IL-17RA, did not show any clinical benefits for patients (199). However, a subpopulation within the trial cohort did show a clinically meaningful change in bronchodilator reversibility. Similarly, as a number of clinical and experimental studies have implicated the inflammasome and IL-1 mediated responses in neutrophilic asthma and steroid-resistance (50, 195, 200, 201), the notion of therapeutically targeting IL-1β has progressed to clinical trials (202). Although use of an IL-1 receptor agonist reduced IL-1β, IL-6, and IL-8 sputum levels in healthy volunteers challenged with LPS (203), trial outcomes in chronic respiratory disease have not yet been conclusive.

Due to the multifaceted nature of asthma, it is likely that treatment will be more successful using a personalized medicine approach. To achieve this, we must first understand whether the measured inflammation is a cause of disease or is a consequence of other underlying pathology not yet understood. This is perhaps exemplified by trials targeting the neutrophil chemokine receptor CXCR2. Although CXCR2 antagonists reduced neutrophil levels in sputum and blood, they did not reduce exacerbations, improve lung function or asthma score. Furthermore, neutrophil levels were reversible and was reversible after treatment ceased (204, 205). Identifying the underlying mechanisms in asthma pathogenesis will likely uncover treatable traits to develop novel therapeutics or reveal stratification strategies using phenotypic traits or biomarkers to more effectively treat patients with currently available therapies using a more targeted approach.

As accumulating evidence links respiratory tract infections and asthma pathogenesis, reducing the burden and carriage of the implicated pathogens has been suggested as an alternate strategy. Here we discuss some potential alternate and novel therapeutic strategies.

### Vaccination

The annual influenza vaccine is available for individuals with asthma; however, no vaccine is currently available for RSV, RV, Mcat, or NTHi. Promisingly, vaccine candidates for these pathogens using a variety of vaccine technologies are at various stages of development (206–209), but further clinical trials are needed to assess their efficacy in reducing exacerbations in chronic respiratory disease. Recently, mRNA vaccines have proposed as an alternative vaccine platform to conventional vaccines (210). The success of the SARS-CoV-2 mRNA vaccine potentially signifies the beginning of a new era of vaccination technology, which may allow for development of vaccines against respiratory pathogens previously difficult to vaccinate against (211).

Other alternative vaccinology methods such as Trained Immunity-based Vaccines (TIbV) have also been proposed. These vaccines are composed of multiple microbial components aimed at generating broad responses and stimulation of trained immunity to promote immunotolerant responses to live pathogens (212). Generating protective immune responses is also the aim of bacterial lysate therapy. Roth et al. (213) found that treatment with bacterial lysate components (OM-85) inhibited RV infection of airway epithelial cells *in vitro*, through increased anti-viral activity and upregulation of proteins involved in antigen presentation. A study using a murine infection model has also shown beneficial effects of bacterial lysate treatment on RSV and influenza infection, with inhibition of infection suggested to occur through TLR signaling (214). Importantly, bacterial lysate therapy was shown to reduce exacerbation frequency in individuals with asthma (215).

### Novel Antibiotics/Anti-Inflammatories

Although antibiotics are commonly used to treat respiratory infections, antibiotic treatment is non-specific and subsequently has global, detrimental effects on microbiome community structure. Antibiotic use in early childhood may predispose individuals to asthma development by increasing the abundance of bacterial species associated with asthma development (177, 216, 217). However, the AMAZES (Asthma and Macrolides: The Azithromycin Efficacy and Safety) trial identified that azithromycin treatment reduced *H. influenzae* load and exacerbation risk. This trial demonstrated that long term treatment of asthmatic individuals with the macrolide, azithromycin, reduced exacerbations (218), and *H. influenzae* load in the airway (219). This finding was consistent with an earlier study in children with bronchiectasis treated with azithromycin (220). However, both studies found increased carriage of antibiotic resistance genes, a concern given the current antimicrobial resistance crisis. As such, reducing *H. influenzae* load through macrolide therapy may prevent exacerbations and the development of steroid-resistance in later life, but antibiotic use in early life may cause more detrimental effects.

Macrolide antibiotics have immunomodulatory properties, as well as antibacterial, which could improve the ability of macrophages to respond to NTHi in the airway. Use of another macrolide antibiotic, clarithromycin, reduced IL-17 responses in a murine model of *H. influenzae*-induced severe, steroid-insensitive, neutrophilic allergic airways disease (221). One of the suggested immunomodulatory properties of macrolides is promotion of macrophage phagocytosis. Treatment of healthy primary human AM and THP-1 differentiated macrophages with two novel non-antibiotic macrolides *in vitro* resulted in increased phagocytosis of NTHi and apoptotic epithelial cells, decreased IL-1β levels and inflammasome activation (222). However, as macrophage phagocytosis decreases with worsening disease severity (76), the mechanisms of impairment may differ between disease states and phagocytosis may not be restored by macrolide treatment. Use of *ex vivo* models may better recapitulate the human lung environment when assessing treatment efficacy of novel therapeutics (223). Nonetheless, the benefit of reducing *Haemophilus* presence in the airway is clear, highlighting the importance of discovering alternative, non-antibiotic avenues of therapy which can enhance or restore immune cell function, minimize airway bacterial load, and reduce inflammation.

### Restoring the Balance of Host Immunity

The soluble human lung proteins SP-A and SP-D have diverse roles against viral and bacterial pathogens, as well as modulating the immune system and could also be exploited as exogenous therapeutics to treat asthma exacerbations through these mechanisms (224–227). Recombinant versions of these proteins have been developed and could have therapeutic potential in treating asthma exacerbations (225, 228). A recombinant fragment of SP-D is currently been taken forward to a first-in-human trial to prevent neonatal chronic lung disease in preterm infants (224).

As mentioned previously, elevated levels of inflammasome and IL-1 responses have been detected in neutrophilic asthma, but have also been associated with NTHi (50). Associations between NTHi and IL-1β are not limited to asthma (50, 51, 197), with higher levels of IL-1β measured in BAL samples from NTHi+ COPD patients compared to NTHi- COPD patients (229), as well as increased neutrophils in the airways of COPD patients colonized with *H. influenzae* (230). As such, targeting the IL-1β pathway in chronic respiratory disease could attenuate the chronic inflammation caused by persistent NTHi colonization. More work is required to determine whether upregulation of inflammasome responses and IL-1β in chronic respiratory disease is NTHi-specific or pan-bacterial, as IL-1 pathway interventions may only benefit a subset of patients with airway inflammation associated with NTHi-mediated IL-1 pathway activation. Thus, future trials could benefit from stratifying patients based on NTHi presence, which could improve the efficacy of IL-1 therapies that so far have shown to be ineffective in asthma (231).

As anti-viral immunity is impaired in asthma, restoring this immunity may reduce the risk of infection-driven exacerbations. In particular, type I IFN responses, mediated by IFN-β has shown to be crucial in anti-viral immunity (232–234). A randomized controlled trial investigating the effect of inhaled IFN-β did not meet its primary endpoint of assessing asthma symptoms, but did find improvements in morning peak flow and enhanced innate immunity, specifically ISG expression (235). The INEXAS (A Study in Asthma Patients to Evaluate Efficacy, Safety, and Tolerability of 14 Days Once Daily Inhaled Interferon Beta-1a after the Onset of Symptoms of an Upper Respiratory Tract Infection for the Prevention of Severe Exacerbations) trial also found small improvements in morning peak flow following use of inhaled IFN-β, but again, the primary endpoint was not met and the impact of inhaled IFN-β on the rate of severe asthma exacerbations was unable to be assessed (236). In contrast, inhaled IFN-β therapy was found to increase the odds of improvement in clinical status as well as time to recovery for hospitalized COVID-19 patients (237). Importantly, studying the dynamics of IFN responses found that timing of IFN-β dosing is key; prophylactic IFN-β treatment reduced influenza infection of macrophage and epithelial cells, whereas this was not observed if cells were treated with IFN-β after influenza infection (238). Thus, the timing of treatment initiation is crucial for disease outcomes in those with underlying dysregulated immune responses, which will undoubtedly guide the development of future clinical studies and treatment guidance for individuals suffering from viral-induced exacerbations.

### Microbiome Modulation: A Feasible Alternative Therapeutic Approach?

The accumulating number of studies demonstrating the presence of potentially pathogenic bacteria preceding viral infection and influencing respiratory health and asthma development, indicate that perhaps targeting these bacteria may be an attractive alternative therapeutic approach. Conversely, the absence of commensal bacteria may contribute to impaired immune development and training (239, 240). As such, attention turns to determining whether promoting the restoration of respiratory tract commensal species may result in more favorable outcomes, as has been observed for probiotics and the gut microbiome (241).

The importance of the commensal bacteria members of the microbiome for development of efficient immune responses has been previously shown in murine models. Antibiotic-treated mice displayed altered immune responses to respiratory viral infection following depletion of commensal bacteria (239, 240). The importance of microbiome community structure for immune training and development in the airway during early life is also shown in germ-free mice, who develop an exaggerated response to allergen challenge in the absence of microbial colonization (242). Furthermore, specific lung bacteria were either protective or inductive of certain asthma features following inoculation of mice, highlighting the divergent immunostimulatory capacity of different bacteria (243). These murine studies demonstrate the importance of the microbiome community structure in shaping appropriate immune responses to both allergens and infection.

Identification of the bacteria important for immune training and stable respiratory health is crucial if modulation of the lung microbiome is to prove feasible. Bosch et al. found that certain children transitioned more quickly from a *Staphylococcus*-profile to a *Moraxella-*dominant profile, bypassing a C*orynebacterium/Dolosigranulum*-dominated profile. Prolonged presence of this latter profile was associated with fewer RTIs and timely maturation of the microbiome in healthy children (178). This is in agreement with an earlier study by Biesbroek et al., who similarly identified C*orynebacterium/Dolosigranulum* airway presence to be associated with decreased number of parental-reported upper RTIs (169). A potential mechanism was suggested following modeling work, which inoculated infant mice intranasally with C*orynebacterium pseudodiphtheriticum*. Activation of TLR3-mediated antiviral immunity was detected, resulting in reduced lung RSV viral titers and reduced susceptibility to secondary bacterial infection (244). Importantly, non-viable *C. pseudodiphtheriticum* did not induce the same protective responses, indicating the importance of live *C. pseudodiphtheriticum* colonization for modulating host immune responses.

Human challenge models have also shown the beneficial, protective effects of colonizing commensal bacteria; inoculation of human volunteers with *Neisseria lactamica* reduced *N. meningitidis* carriage (245). It is clear that the presence or absence of certain microbial species and their functional microbial interactions influence disease susceptibility and trajectory and unbalance host immune responses. However, as a recent study by Thorsen et al. implicates potential commensal *Prevotella* with altered host responses and asthma development, it is important to first ascertain the pathogenic potential of microbes when considering therapeutically altering microbiome structure (246). Nonetheless, unraveling the complex interplay between infection, atopy, host immunity, and genetic factors in early life will provide novel insights and may allow advanced identification and stratification of individuals at risk of developing asthma, for example if they possess certain genetic risk factors and microbiome characteristics.

Although the role of bacteria in asthma is only now becoming appreciated, there are even fewer studies investigating the role of fungi and the mycobiome in health and disease (247). Recent inclusion of fungi in airway microbiome studies has revealed a distinct airway mycobiome which is altered in asthma (248). A role for fungi in early life asthma development has also been suggested by Stern et al., who found that sensitization to certain fungal species was associated with asthma in later life (249). A recent study has identified associations between *Moraxella* presence and asthma-associated fungi (250). Given the possibility for multiple microbiota-host and other environmental interactions to occur, future studies need to ensure that all potential contributing factors are accounted for in study design and conclusions, in order to develop effective therapeutics.

## Summary

Accumulating evidence indicates an important role of both bacteria and viruses in driving asthma pathogenesis. Although viruses have long been implicated in asthma development and exacerbations, the enrichment of certain bacteria in the airway appears to play a more prominent role in asthma pathogenesis than initially believed. The composition of the microbiome in early life is crucial for immune training and development, which is reflected by aberrant responses in later life. Omics technologies are beginning to reveal the extent of microbiome modulation of the host in the respiratory tract and understand how it influences host-bacteria-virus crosstalk and relates to disease severity and progression. Although current and developing therapies aim to reduce neutrophil/eosinophil recruitment and activation in asthma, emerging evidence indicates that the microbiome may contribute to chronic and dysregulated airway inflammation. As such, microbiome modulation may instead be an attractive alternative for managing airway inflammation and host immunity. This approach may rebalance and retrain appropriate host immune responses to inflammatory triggers and subsequently reduce the risk of asthma development in those with genetic predispositions. Furthermore, it may reduce the risk of exacerbation and progression to more severe disease in those with asthma already established.

## Author Contributions

JA, KS, and TW conceptualized the review article. JA and KS wrote the original draft. JA performed the literature search. AW and TW reviewed and edited the manuscript. KS and TW supervised. All authors approved the final version of the manuscript.

## Funding

This work was funded by an Asthma UK studentship award (AUK-PHD-2016-363).

## Conflict of Interest

KS reports grants from AstraZeneca outside the conduct of the study. TW reports grants and personal fees from AstraZeneca, personal fees and other from MMH, grants and personal fees from GSK, grants and personal fees from AZ, personal fees from BI, grants and personal fees from Synairgen, outside the submitted work. The remaining authors declare that the research was conducted in the absence of any commercial or financial relationships that could be construed as a potential conflict of interest.

## Publisher's Note

All claims expressed in this article are solely those of the authors and do not necessarily represent those of their affiliated organizations, or those of the publisher, the editors and the reviewers. Any product that may be evaluated in this article, or claim that may be made by its manufacturer, is not guaranteed or endorsed by the publisher.
